# Simulation-based team training in time-critical clinical presentations in emergency medicine and critical care: a review of the literature

**DOI:** 10.1186/s41077-021-00154-4

**Published:** 2021-01-20

**Authors:** Jesper Weile, Mette Amalie Nebsbjerg, Stig Holm Ovesen, Charlotte Paltved, Mads Lind Ingeman

**Affiliations:** 1 Emergency Department, Regional Hospital Horsens, Horsens, Denmark; 2 Research Center for Emergency Medicine, Aarhus University Hospital, Palle Juul-Jensens Blvd. 161, 8200 Aarhus, Denmark; 3 Department of Internal Medicine, Regional Hospital West Jutland, Herning, Denmark; 4 Corporate HR MidtSim, Central Denmark Region, Aarhus, Denmark; 5 Department of Emergency Medicine, Aarhus University Hospital, Aarhus, Denmark

**Keywords:** Emergency medicine, Critical care, Simulation-based team training

## Abstract

**Background:**

The use of simulation-based team training has increased over the past decades. Simulation-based team training within emergency medicine and critical care contexts is best known for its use by trauma teams and teams involved in cardiac arrest. In the domain of emergency medicine, simulation-based team training is also used for other typical time-critical clinical presentations. We aimed to review the existing literature and current state of evidence pertaining to non-technical skills obtained via simulation-based team training in emergency medicine and critical care contexts, excluding trauma and cardiac arrest contexts.

**Methods:**

This systematic review was conducted in accordance with the Preferred Reporting Items for Systematic Reviews and Meta-Analysis (PRISMA) statement. Before the initiation of the study, the protocol was registered in the International Prospective Register of Systematic Reviews (PROSPERO) database. We conducted a systematic literature search of 10 years of publications, up to December 17, 2019, in the following databases: PubMed/MEDLINE, EMBASE, Cochrane Library, and CINAHL. Two authors independently reviewed all the studies and extracted data.

**Results:**

Of the 456 studies screened, 29 trials were subjected to full-text review, and 13 studies were included in the final review. None of the studies was randomized controlled trials, and no studies compared simulation training to different modalities of training. Studies were heterogeneous; they applied simulation-training concepts of different durations and intensities and used different outcome measures for non-technical skills. Two studies reached Kirkpatrick level 3. Out of the remaining 11 studies, nine reached Kirkpatrick level 2, and two reached Kirkpatrick level 1.

**Conclusions:**

The literature on simulation-based team training in emergency medicine is heterogeneous and sparse, but somewhat supports the hypothesis that simulation-based team training is beneficial to teams’ knowledge and attitudes toward non-technical skills (Kirkpatrick level 2). Randomized trials are called for to clarify the effect of simulation compared to other modalities of team training. Future research should focus on the transfer of skills and investigate improvements in patient outcomes (Kirkpatrick level 4).

**Supplementary Information:**

The online version contains supplementary material available at 10.1186/s41077-021-00154-4.

## Background

The use of simulation-based team training as an educational tool has increased over the past decades [1, 2]. Healthcare professionals most often welcome simulation training, and team-based simulation has been shown to improve knowledge, skills, and behaviors [3, 4]. In emergency medicine (EM), simulation-based team training is best known for its use in trauma and cardiac arrest team training. However, many other patients require time-critical management, including patients in shock, suffering from hypoglycemia, or experiencing adrenal crisis, where a team-based approach is desirable [4].

The purpose of simulation-based training is ultimately to increase the quality of patient treatment and patient safety by increasing adherence to the principles of a team-based approach to resuscitation [5]. If the team adheres to these principles, this will in theory optimize time-critical diagnostics and interventions [5]. If critical diagnostics and time-critical interventions are optimized, it has been speculated that this will improve important patient outcomes, such as morbidity and mortality [6].

The Kirkpatrick Model is well-known as a tool for analyzing and evaluating the results of education and training. It presents a hierarchy that stratifies the level of impact of training results according to four levels: clinical parameters and patient outcomes (level 4), changes in the participants’ behavior after training (level 3), individual learning (level 2), and participants’ initial reactions toward training (level 1) [7].

The focus and results of simulation-based training consist of technical and non-technical skills. Technical skills are defined as the “adequacy of the actions taken from a medical and technical perspective,” while non-technical skills are defined as the “decision-making and team interaction processes used during the team’s management of a situation” [8, 9]. Per definition, training in non-technical skills requires a team effort, whereas technical skills can be performed and measured at either the individual or the team level [10, 11].

Simulation-based training can be performed either in situ or in a simulation center. In situ training takes place in the clinical environment where patients are usually received, and the participants are the actual staff on call; this is the antithesis of training that occurs in a facility away from the clinical setting, in which participants practice team training on a course or in a simulation training program [12]. Simulation mannequins or actors can be used in both settings, and the simulation mannequins can have a variety of patient-like features, including voices and reacting pupils. Depending on the complexity of the set-up, simulations can be denominated either high fidelity or low fidelity [13].

Original research on and reviews of simulation-based team training with trauma or cardiac-arrest teams is manifold [14–16]. It has been concluded that simulation-based team training contributed to a significant effect on learning within trauma training [16] and that simulation-based team training contributes to increase in survival to discharge when training in cardiac arrest was implemented [17]. However, research into simulation-based training in other time-critical clinical presentations is not gaining the same attention. It has been proposed to view the obtained experience from simulation-based team training as a library to draw upon when specific scenarios are encountered [18]. It is important to acknowledge that many time-critical diseases other than traumas and cardiac arrest are present in everyday emergency medicine and critical care. These are also taught in simulation-based team training. Hence, broadening simulation training beyond cardiac arrest and trauma is important. Cardiac arrest and trauma care consists of multiple case-dependent tasks (i.e., compressions of the chest), often involve larger and multispecialty teams, and are founded on uniform training prerequisites. Therefore, emphasis on simulation training outside these realms is important. However, the necessary evidence must exist before venturing into costly simulation-based team training. No recent reviews have focused solely on simulation-based team training in EM and critical care outside the trauma team and cardiac arrest contexts.

Since simulation-based team in training cardiac arrest and trauma are covered elsewhere and much training is beyond these areas, we aim to review the existing literature on simulation-based team training in EM and critical care in time-critical patient presentations. The research question for this review is as follows: What literature exists on simulation-based team training within emergency medicine and critical care outside cardiac arrest and trauma? Secondarily, we ask what kinds of training have been researched within this delimitation.

## Methods

### Review questions

This review aimed to determine what evidence exists to support the usefulness of simulation-based team training in EM and critical care regarding the following:

- Improvement of attitudes toward simulation training (Kirkpatrick Model level 1)
- Improvement of team skills in simulation settings (Kirkpatrick Model level 2)
- Improvement of team skills in clinical practice (Kirkpatrick Model level 3)
- Improvement of clinical parameters and patient outcomes (Kirkpatrick Model level 4)

The Preferred Reporting Items for Systematic Reviews and Meta-Analysis (PRISMA) statement was used for the reporting in this review.

### Protocol and registration

The details of the protocol for this systematic review were registered in the International Prospective Register of Systematic Reviews (PROSPERO) before title and abstract screening began (Record ID: 161941).

### Eligibility criteria

Study inclusion adhered to the following population, intervention, comparison, and outcomes (PICO) criteria:

- Population: We aimed to identify studies working with populations of health care providers (nurses, doctors, technicians, paramedics, and so on) with clinical responsibilities in EM and critical care.
- Intervention: We aimed to identify studies examining simulation-based team training in EM and critical care (in situ or in a simulation center).
- Comparison: We aimed to identify studies comparing participant performance before (control) versus after (intervention) simulation-based team training. The control group was permitted to have been training or teaching as usual or to have undergone no training at all. Participant evaluations occurred over time. We also accepted comparisons between pre- and post-intervention outcomes.
- Outcomes: We aimed to identify studies investigating the following: (1) improvement of clinical parameters and patient outcomes; (2) improvement of non-technical skills in clinical practice; (3) improvement of non-technical skills in the simulation setting; and (4) improvement in attitudes toward simulation training.

We included all published studies within 10 years prior to the search dates (December 16 and 17, 2019). Manuscripts in English, Danish, Swedish, and Norwegian were included. All study designs were included. We excluded the following types of studies: (1) studies on undergraduate team training; (2) studies focusing exclusively on technical skills; (3) teams with less than three people or without at least one physician on the team; (4) studies focused exclusively on cardiac arrest and/or trauma teams; (5) studies focused exclusively on pediatric patients; and (6) studies focused exclusively on surgical emergencies.

### Information sources

We searched the following databases: PubMed/MEDLINE, EMBASE, Cochrane Library, and CINAHL. Citations from included manuscripts were screened, and relevant studies were included in the review.

### Literature search

Search terms included the following: Crisis Intervention, Crisis Resource Management, Advanced Life Support, Emergency Medicine, Critical Care, Patient Care Team, Interprofessional Relations, Interdisciplinary Team, Medical Emergency Team OR Medical Emergency Response Team, Simulation Training, Simulation Based. The full search protocol for all databases is available in Supplement 1. An experienced librarian performed the search.

### Study selection

MN and JW independently performed the title and abstract screening for eligibility. MN and JW subsequently screened eligible studies via full-text reading for inclusion. The study inclusion process was performed using the Covidence software platform (Covidence.org). In cases of disagreement, a third reviewer (MI) was consulted to obtain consensus.

### Data collection process and data items

MN, JW, and SH extracted pre-specified data from each included study. The data included first author, year, country/countries, study design, aim, intervention, control group, type of participant(s), in situ/simulation center, fidelity level, re-test, outcome measure, main results, and Kirkpatrick level.

MN, SH, and JW assessed the risk of bias for the included studies. According to the study design, the following risk of bias tools were used:

- Non-randomized trials: Risk of Bias in Non-Randomized Studies of Interventions (ROBINS-I) [19].
- Qualitative studies: Critical Appraisal Skills Programme (CASP) Score [20].

Due to the heterogeneous outcome measures, no meta-analysis was performed. Facing the inability to conduct meta-analysis, we conducted a narrative synthesis of the results from the included studies. We performed the synthesis following the guidelines proposed by Popay et al. [21].

## Results

### Study selection

**Fig. 1:**
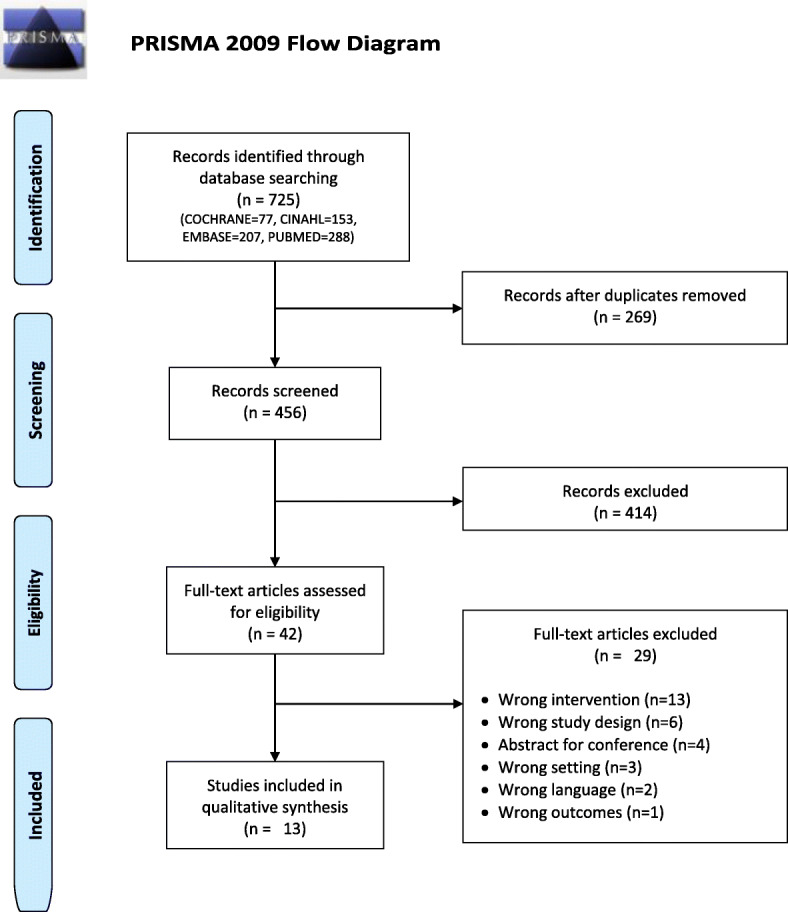
PRISMA flow diagram of the included studies

The search identified 725 studies and yielded 456 unique studies when duplicates were removed. After title screening and abstract screening, respectively, 414 and 29 studies were identified for full-text screening. A total of 13 full-text studies were included for data extraction and synthesis in this review. Figure 1 shows the PRISMA flow diagram.

### Study characteristics

Further details on the included study designs, participants, and Kirkpatrick levels are presented in Table 1. Further details on the study aims, interventions, simulation characteristics, outcomes, and results are presented later in Table 2. None of the studies were randomized controlled trials.

**Table 1:** Included studies and sample size/types of participants and the Kirkpatrick level investigated in each study

Study	Country	Study design	Sample size/type of participants			Kirkpatrick level				
			Physicians	Nurses	Other		I	II	III	
Bond et al. [22]	Chicago, USA	Pre-post	9	42	9 ED technicians			X		
Brewster et al. [23]	Australia	Pre-post	19	80				X		
Chan et al. [24]	Hong Kong, China	Pre-post	26	46				X		
Couto et al. [25]	Sao Paulo, Brazil	Prospective observational	40	24	50 nurse technicians			X		
Dagnone et al. [26]	Ontario, Canada	Pre-post	21	8	12 medical students, 1 respiratory therapist, 1 paramedic		X			
Hicks et al. [27]	Toronto, Canada	Pre-post	14				X	X	X	
Marker et al. [28]	Denmark	Interview study	20						X	
Meurling et al. [29]	Sweden	Pre-post	51	75	25 nurse assistants			X		
Paltved et al. [30]	Denmark	Pre-post, needs analysis	9	30				X		
Parsons et al. [31]	Philadelphia, USA	Pre-post	14					X		
Rasmussen et al. [32]	Denmark	Semi-structured interviews	14	3			X			
Truta et al. [33]	Romania	Pre-post	30	40				X		
Wong et al. [34]	USA	Mixed-methods design. Pre-post.	17	11	10 technicians, 13 protective service, 6 advanced practice providers			X

**Table 2:** Study aims, interventions, simulation characteristics, outcomes, and main results

Study	Aim	Intervention	Control group	In situ/ex situ	Fidelity level (low vs high level mannequin)	Re-test (skill retention)	Outcomes measure	Main results
Bond et al. [22]	To share the development of a telehealth solution for shared awareness between the in situ simulation professionals and the ED bedside team caring for the in situ simulated patient, and the eICU nurses participating via telehealth.	3/4 days workshop with in situ simulation. Site A: 3 sim days Site B: 4 sim days	No	In situ	Patient played by an actor and vital signs generated by Laerdal SimMan.	No	Pre-post self-confidence in using telehealth. Pre-post self-confidence in managing ED patients with sepsis.	Telehealth was feasible with the equipment of choice. Pre-post self-confidence in using telehealth increased from a mean ± SD of 5.3 ± 2.9 to 8.9 ± 1.1 (Δ3.5, *p* < 0.05). Self-confidence in managing ED patients with sepsis increased from a mean ± SD of 7.1 ± 2.5 to 8.9 ± 1.1 (Δ 1.8, *p* < 0.05).
Brewster et al. [23]	To show a new inter-professional team-based simulation, ALS training package would improve critical care staff performance, understanding, and satisfaction with ALS training	A 4-h workshop	No	NR	Resusci Anne®Simulator	Questionnaire 4 months after workshop attendance.	Changes in questionnaire scores pre-course, immediately after, and 4 months after simulation training.	Overall ICU nursing attendance increased from 54 to 71%. Nurses gave higher scores for all criteria when assessing the new ALS training program compared to the previous program. No significant improvement in perceptions of team performance.
Chan et al. [24]	To evaluate the attitude of participants and their change in knowledge on clinical performance after attending a new training workshop.	2-day workshop (eight 1-h scenarios)	No	Ex situ	NR	No	Knowledge multiple-choice questionnaire.	Improved knowledge of clinical performance and training was well received.
Couto et al. [25]	To detect LST in a training program, which combined in situ simulation scenarios with just-in-time and just-in-place self-directed task training in an ED.	4 simulation weeks with in situ simulation in a year, 14–15 scenarios per simulation week; 10 min scenario, 10 min debriefing.	No	In situ	High-fidelity mannequin (SimMan 3G, SimBaby or SimNewB, Laerdal)	No	LST detected during debriefing.	The training allowed a high rate of detecting LST regardless of theme. Equipment-related LST were more frequently found.
Dagnone et al. [26]	To share the development and evaluation of a simulation-based competition.	3-day simulation-based competition, 3–4 scenarios per team.	No	Ex situ	Trained standardized actors	No	Characteristic of participants, their attitudes toward simulation, and their evaluation of the competition.	Participants were extremely satisfied with the event and expressed a strong desire to expand interdisciplinary team training in resuscitation.
Hicks et al. [27]	To evaluate the feasibility of a simulation-based CRM curriculum for EM residents and identify shifts in team-based behaviors and attitudes.	Precourse learning and 1-day course using simulated resuscitation scenarios paired with focused debriefing sessions. Four scenarios in total.	No	Ex situ	Two high-fidelity scenarios and two low-fidelity scenarios	No	Pre/post scenarios were scored using Ottawa CRM GRS. Pre-post survey on human factor attitude.	Quality, relevance, and potential impact of training were highly positive. Improvement in Ottawa GRS, but not statistically significant from pre- to post-course.
Marker et al. [28]	To identify first-year doctors’ perceptions, reactions, and reflection on transfer of skills after simulation-based training	4-days simulation-based training.	No	Ex situ	High-fidelity	No	Interviews	Increased preparedness. Useful algorithms. Better communication and teamwork.
Meurling et al. [29]	To explore differential individual training effects for physicians, nurses, and nurse assistants on self-efficacy and experienced quality of collaboration and communication between professionals	4 h of interactive seminars concerning safe teamwork. 1 day of simulation training, with a team comprising 6 persons. Each team experienced 3–4 scenarios.	Yes	In situ	High-fidelity	No	Self-efficacy questionnaire. SAQ. Staff turnover and sick leave.	Self-efficacy: The effect for women was 0.21 (95% CI 0.039 to 0.371) and for men 0.59 (95% CI 0.308 to 0.876). SAQ: Discrepant attitudes about teamwork between physicians and nurses. The scores for safety climate improved for nurses. Physicians did not change in scores. Sick leave: Nurse assistants decreased their sick leave from 28 to 12%.
Paltved e t al [30].	To enhance patient safety attitudes through the design of an in situ simulation program based on a needs analysis involving thematic analysis of patient safety data and short-term ethnography.	One scenario, 2 h per team (45-min scenario, 50-min debriefing)	No	In situ	Simulated patients	No	SAQ and Trainee Reactions Score	An in situ simulation program can act as a significant catalyst for improvement in emergency staff’s safety and teamwork attitudes that might correlate with a more positive patient safety culture.
Parsons et al. [31]	To design a CRM course for ED residents and to test the course’s efficacy.	½-h lecture followed by 6 simulation scenarios, 3 active and 3 observed. Scenarios of 15 min with 30 min debrief.	No	Ex situ	High-fidelity (actor or SimMan3G)	No	Ottawa CRM GRS.	Increase in score concerning leadership, problem solving, situational awareness, resource utilization, and communication. Not statistically significant.
Rasmussen et al. [32]	To identify long-term intended and unintended learner reactions, experiences, and reflections after attending a simulation-based ALS course.	ALS course. Duration NR	No	Ex situ	NR	No	Interviews	“(…) the efficiency dimension of ALS competence is taught well in ALS courses, but that the form and content of these highly structured/model courses are insufficient in training the innovative dimension of competence that is needed for transfer of skills in unstructured, emergency situations.”
Truta et al. [33]	To assess whether a CRM-oriented team training combining didactic and simulation sessions improves interprofessional EM team performance of non-technical skills.	1-day (6–7 h) lecture and 6 scenarios (3 active + 3 observed)	No	In situ and ex situ	High-fidelity manikin	Post-test 2 months after intervention	Scale from Flowerdew et al [35].	Improvement in management and supervision, teamwork and cooperation, decision-making, and situational awareness. Statistically significant improvements in all groups of participants.
Wong et al. [34]	To investigate agitation care delivery and to evaluate the impact of a team-based simulation on ED staff.	2-h course, 15 min simulation, focus group interview	No	In situ	Low-fidelity	No	KidSIM ATTITUDES questionnaire. Uniprofessional and interprefessional focus group interviews.	KidSIM: Improvements in attitudinal scores for all questions within the relevance of simulation and opportunities for interprofessional education constructs (all *p* < 0.001). Interviews: The interprofessional conversations fostered insightful discussions regarding the development of novel team-based strategies and solutions for improved agitation management.

*NR* not relevant, *ED* emergency department, *EM* emergency medicine, *eICU*, *ALS* advanced life support, *LST* latent safety threats, *CRM* crisis resource management, *GRS* Global Rating Scale, *SAQ* Safety Attitudes Questionnaire

### Participants

According to the inclusion criteria, all studies involved physicians and teams. The included number of participants ranged from 14 to 151 with a median of 57. The number of included physicians ranged from 9 to 51 (median 19). Besides physicians, 10 studies included nurses, ranging from 3 to 75 (median 27), 5 studies included technicians or nurse assistants, ranging from 1 to 50 (median 10), 1 study included 1 paramedic, and 2 studies included advanced practice providers, with 1 study numbering 6 and the other an unknown number less than 9 [22].

### Interventions

The studies used heterogeneous interventions. However, all studies included team-based simulation training. The length of training ranged from 15 min to 4 weeks (median 1 day); 1 study did not report the time of training. The use of a combination of didactic sessions and simulation-based training was reported in three studies. In situ simulation was used in 7 out of 13 studies, and eight studies used high-fidelity mannequins in the simulation training.

### Comparison

The majority (*n* = 9) of studies used a pre-post design without a control group [22–24, 27, 29–31, 33]. Only one study included a retention test [33].

### Outcomes

All studies used outcomes that could be stratified according to the Kirkpatrick Model. No studies investigated the impact of training on patient outcomes, such as length of hospital stay or mortality, and hence, the transfer of learning to patient outcomes has not been investigated in any study. Two studies measured behavioral changes in professional settings, and hence reached Kirkpatrick level 3 [27, 28]. To assess participants’ changes in behavior, two studies used the validated Ottawa Global Rating Scale [36]. Out of the remaining 11 studies, 9 reached Kirkpatrick level 2, and only 2 reached level 1. To assess changes in attitudes toward patient safety, two studies used the validated Safety Attitudes Questionnaire [29, 30].

### Main results

Below is a short summary of the studies revealed in this review. Overall, 10 quantitative studies and 3 qualitative studies were identified.

#### Kirkpatrick level 3

The two studies that reached the highest Kirkpatrick level were Hicks et al. [27] and Marker et al. [28]. Hicks et al. reported pre- and post-test results on levels 1, 2, and 3 [27]. The intervention was a 1-day course with 14 participating residents, out of which 10 residents participated in pre- and post-course simulations. Results on Kirkpatrick level 1 were collected via a post-course survey, in which residents agreed or disagreed with predefined statements. Interdisciplinary team training was endorsed by all participants, and agreement was reached regarding the positive impact of the training. Kirkpatrick level 2 was assessed using the Human Factors Attitude Survey (HFAS), which can assess attitudinal shifts regarding team behaviors [37]. The participants filled out the HFAS before and after the intervention. Only 1 out of 23 statements had a statistically significant positive change. To assess Kirkpatrick level 3, the Ottawa Crisis Resource Management Global Rating Scale (Ottawa GRS) was used by two independent reviewers. The reviews were performed using DVD film clips of the pre- and post-course simulations. The Ottawa GRS is a behavioral assessment tool that has been proven valid and has a high interrater reliability [36, 38]. Hicks et al. found a tendency toward better performance in the post-intervention simulation. However, no statistically significant changes were reported between the pre-test and post-test scores. The authors concluded that the lack of significance could be a result of underpower.

Marker et al. [28] reported improvement in preparedness, communication, and teamwork after an intervention when compared to before the intervention. Their results were based on post-intervention interviews with participating physicians. Hence, their results reflected the subjective evaluations of the physicians. The participating physicians described incidents in which the simulation course had changed their behaviors in their everyday clinical lives in a positive direction. No objective scales were used in this trial, which was purely qualitative.

#### Kirkpatrick levels 1 and 2

On the two lowest Kirkpatrick levels, there were multiple studies using both quantitative and qualitative designs.

One of the studies used a control group. In this study by Meurling et al. [29], a secondary outcome was a reduction in sick leave when a simulation-training program was introduced in an intensive care unit (ICU). To measure the outcome, a different ICU from the same hospital was used as a control. The result was positive and found a decrease in sick leave in the intervention group and an increase in sick leave in the control group during the study period. Meurling et al. [29] also reported changes in self-efficacy identifiable between questionnaires given before and after the training. On a 1–7-point Likert scale, they found a significant rise from 5.6 (SD = 0.9) to 5.9 (SD = 0.7); *p* < 0.0001.

Similar to Hicks et al. [27], video-recorded simulations were used by Parsons et al. [31] and Truta et al. [33]. Both studies used recordings from a test simulation of all participants before and after the intervention. In both studies, two observers reviewed the recordings, and the observations were rated on a global rating scale. Parsons et al. evaluated a cohort of 14 EM interns on leadership, problem solving, situational awareness, resource utilization, and communication, as well as providing an overall performance score, using the Ottawa CRM Global Rating Scale (GRS) [36]. The authors found no statistically significant improvements resulting from the intervention and concluded that their study was underpowered. Truta et al. [33] had two blinded observers assess the skills of 30 physicians and found statistically significant improvement in the following measured modalities: management and supervision, teamwork and cooperation, decision-making, and situational awareness.

Bond et al. [22] aimed to investigate the benefit of tele-health communication assistance provided by an ICU nurse during simulation. The introduction of a bidirectional video cart made it possible to consult an experienced ICU nurse during an in situ simulation of septic shock. The outcome was self-reported self-confidence before and after the intervention. The results revealed a rise in self-confidence in managing sepsis from a mean of 7.1 (SD = 2.5) to 8.9 (SD = 1.1); *p* < 0.05.

Brewster et al. [23] also used questionnaires before and after an intervention. The intervention consisted of pre-course learning and 4 h of lectures and simulation. The results were based on questionnaires that were supplemented by a third questionnaire 4 months after the intervention, hence reaching Kirkpatrick level 2. The study revealed a statistically significant rise in satisfaction among nurses and physicians before and after the intervention.

One study found a different approach to simulation training. Dagnone et al. [26] presented their results after conducting “simulation Olympics” (i.e., an intervention in which 11 teams competed against each other in simulated resuscitation scenarios). The study investigated the participants’ responses to an evaluation of the Olympics and found that all participants but one “strongly agreed” or “agreed” to statements expressing satisfaction with the event.

Chan et al. [24] investigated simulation training and relied on questionnaires. They found an increase in scores on a multiple-choice questionnaire for questions regarding resuscitation. The improvement between mean pre- and post-test scores was 11.5%. Couto et al. [25] investigated the use of simulation to improve participants’ ability to identify latent safety threats (LST). An observer filled out a checklist during debriefing and found a higher proportion of equipment-related LST (*p* < 0.01) after the intervention. These two studies were assessed to reach Kirkpatrick level 2.

Three studies had study designs differing from the aforementioned studies. The first study, by Wong et al. [34], used a mixed-methods approach to investigate the usefulness of interprofessional standardized patient simulation for emergency-department agitation management. An actor who received instructions through an earpiece during the simulation played a standardized patient. All participants were interviewed in interprofessional and uniprofessional focus groups after the intervention. The interviews revealed a “need and desire for more interprofessional training in agitated patient care.”

In a mixed-methods study by Paltved et al. [30], results were presented from a thematic analysis, a needs analysis, and an evaluation of a simulation-training program. The authors argued that a needs analysis is required in order to tailor training for emergency departments. The needs analysis revealed that the handover between shifts could be frustrating and that a common language is lacking. A result of this was that the “staff valued clear and structured communication and communication strategies such as thinking aloud in order to enhance patient safety. These communication skills improved shared understanding, but interruptions impaired communication.” Furthermore, the study included a validated questionnaire (the Safety Attitudes Questionnaire [SAQ]) filled out before and after simulation training, which revealed an increase in the safety climate from the pre-SAQ score (mean = 25.74, SD = 4.41) to the post-SAQ score (mean = 26.59, SD = 4.23); *p* < 0.001.

Lastly, Rasmussen et al. [32] conducted 17 semi-structured telephone interviews with personnel who had participated in an Advanced Life Support (ALS) course. They analyzed the data using a constructivist grounded theory approach and found challenges transferring the skills from the course to the clinical setting. This was mostly due to the fact that other personnel in the clinical setting did not have the same course background. They concluded that the course was insufficient in training for the development of transferable skills.

### Risk of bias within studies

**Fig. 2:**
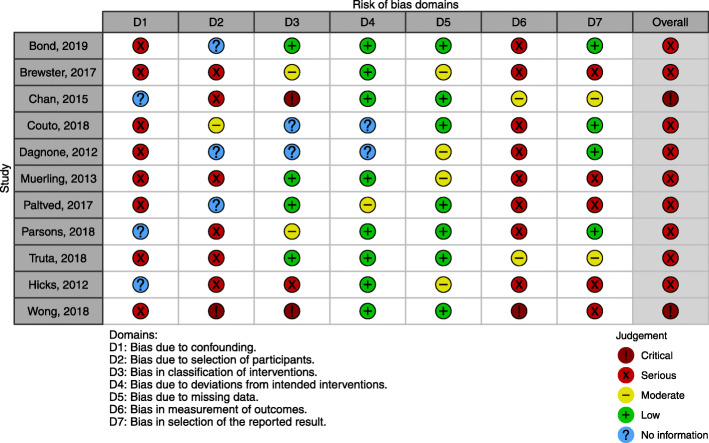
Risk of bias summary in quantitative studies

All studies in this review were associated with a serious risk of bias. The reporting of important aspects of the studies was incomplete, leaving items unclear. Figure 2 shows the risk of bias assessment for the non-randomized trials using the ROBINS-I tool, and Table 3 shows the risk of bias assessment for the two qualitative studies using CASP [20].

**Table 3:** Risk of bias summary in qualitative studies

CASP scale	Marker et al. [28]	Rasmussen et al. [32]
Clearly focused question	+	+
Appropriate method	+	+
Appropriate design	-	+
Appropriate recruitment	-	-
Appropriate data collection	+	+
Relationship between participant and researcher	+	+
Ethical issues	-	+
Appropriate data analysis	+	+
Statement of findings	+	+
Valuable research	+	-
**Total score**	7	8

## Discussion

This systematic review revealed 13 studies investigating the impact of simulation training within EM and critical care. All studies focused on simulation-based team training outside the realms of trauma and cardiac arrest.

The study conducted by Hicks et al. [27] stands out in terms of its thoroughness and rigidity. Even though the researchers did not find statistically significant results in favor of simulation, the scaffolding of the study could serve as an inspiration for future work in the field of simulation training. We agree with the authors that the study was underpowered, and the participants were not randomly assigned. We encourage future researchers indulging in team-based simulation to follow the framework set forth by this group. Adding a control group to the study would have strengthened the study, as would random allocation of the participants.

The risk of bias was serious in all studies included. Many studies have been performed on existing courses, leaving the authors with little to no influence on the intervention design or the choice of participants. A solution to this is the more complicated and less feasible approach wherein the investigators themselves design the study and ensure the inclusion of participants in a manner that involves little to no risk of inclusion bias. This will in many cases be a more costly approach [39]. However, this investment will lead to the production of more valuable evidence regarding the actual benefit of simulation-based team training.

In general, studies used pre- and post-simulation questionnaires to report the effect of training. Three studies used video recordings to objectify the outcomes. Only a minority of the studies included validated questionnaires like Ottawa GRS and SAQ; this makes a comparison of the studies difficult. We recommend that future studies use objective outcomes like video recordings where possible. When using self-reported outcomes, we recommend the use of validated questionnaires.

The studies included in this review measured outcomes at low Kirkpatrick levels, and no studies reported results on the highest Kirkpatrick levels. This makes it impossible to synthesize measures of transfer of learning to outcomes directly affecting important patient outcomes. Only one study, by Rasmussen et al., described transferability to clinical practice. The course consisted of a pre-course reading preparation and a 2-day mixed course with simulation as well as didactic sessions. The qualitative interview study found the course insufficient for the transfer of skills to a clinical setting [32]. This finding was contrary to what we expected. It remains uncertain whether conducting team-based simulations within EM settings provides competences that can be transferred to clinical settings and thus potentially benefit patients.

The rationale behind the choice of simulation-based intervention is often scarcely described in the studies included in this review. One study, by Paltved et al., conducted a needs analysis to investigate the proper aim of simulation [30]. The studies generally fail to answer the question of whether the same result would have been reached if the training had been longer or shorter or if a different modality of teaching had been chosen. We encourage future studies to thoroughly establish the rationale behind the design of any intervention.

We propose two main directions for future investigations in simulation-based training within EM and critical care. First, randomized trials with outcomes higher than Kirkpatrick 3 are needed. It is paramount for continuing investment in simulation that the actual patient important outcomes, such as morbidity and mortality, be investigated. One costly way to perform such investigations could be multicenter studies where simulation-based team training could be implemented in one half of the involved centers and patient important outcomes could be measured over time. Second, we find that the interventions in the studies in this review are heterogenetic. Research should aim to identify the effects of different interventions rather than comparing an intervention to itself (via pre-post tests). For the benefit of comparing future studies, it will be necessary to provide evidence that investigates the length of the simulation training versus the level of training obtained. As an example, it could be interesting to investigate if 2-day courses are superior compared to 1-day courses.

## Limitations

This review has some limitations. First, the literature review was limited to 10 years from the date of the search. Important studies outside this timeframe may have been missed. Second, the results may have been influenced by publication bias, in that studies with negative results toward simulation have not been published. In this study, this bias may have pushed the results toward a favorable analysis of simulation training.

## Conclusions

The included studies in this review suggest positive outcomes in terms of the benefit of simulation-based team training. However, these studies are associated with a serious risk of bias and report on low levels using the Kirkpatrick Model. Hence, more rigorous research is needed to investigate the evidence of the benefit of team-based simulation in EM and critical care contexts.

## Supplementary Information

**Additional file 1: Supplement 1.** Database searches

## Data Availability

The datasets used and/or analyzed during the current study are available from the corresponding author on reasonable request.

## Abbreviations

PRISMA: Preferred Reporting Items for Systematic Reviews and Meta-Analysis
PROSPERO: Prospective Register of Systematic Reviews
EM: Emergency medicine
PICO: Population, intervention, comparison, and outcomes
CASP: Critical Appraisal Skills Programme
HFAS: Human Factors Attitude Survey
Ottawa GRS: Ottawa Crisis Resource Management Global Rating Scale
LST: Latent safety threats
SAQ: Safety Attitudes Questionnaire
ALS: Advanced Life Support
ROBINS-I: A tool for assessing Risk of Bias in Non-randomized Studies of Interventions
