# Supplementary material for: Simulation-based team training in time-critical clinical presentations in emergency medicine and critical care: a review of the literature
Source: Adv Simul (Lond). 2021 Jan 20;6:3. doi: 10.1186/s41077-021-00154-4 (PMC7816464; doi:10.1186/s41077-021-00154-4)
Supplement: Supplementary file 1 — Additional file 1: Supplement 1. Database searches [file 41077_2021_154_MOESM1_ESM.docx]

**Supplement 1**

**Database searches**

**PubMed 16/12/19**Search: (((("Crisis Intervention"[Mesh]) OR ("Crisis Resource Management" OR "advanced life support" OR "Emergency Medicine"[Mesh:NoExp] OR "Emergency Medicine"[Title/Abstract] OR "Critical Care"[Mesh:NoExp] OR "Critical Care"[Title/Abstract]))) AND ((("Patient Care Team"[Mesh] OR Patient Care Team* OR "Interprofessional Relations"[Mesh] OR Interprofessional Relation*)) OR (team* OR interdisciplinary team* OR medical emergency team* OR medical emergency response team*))) AND ("Simulation Training"[Mesh] OR Simulation based*) Filters: 10 years

**Embase 16/12/19**

**No.**

**Query**

**Results**

**207**

**#17**

**#16** AND (**'article'**/it OR **'article in press'**/it OR **'conference paper'**/it OR **'conference review'**/it OR **'review'**/it)

**412**

**#16**

**#7** AND **#10** AND **#14** AND [2009-2020]/py

**428**

**#15**

**#7** AND **#10** AND **#14**

**362,928**

**#14**

**#11** OR **#12** OR **#13**

**306,677**

**#13**

**'patient care team*'** OR **'interdisciplinary team*'** OR **'medical emergency team*'** OR **'medical emergency response team*'** OR **'interprofessional relations'** OR **team***

**5,205**

**#12**

**'multidisciplinary team'**/exp

**61,693**

**#11**

**'public relations'**/exp

**10,249**

**#10**

**#8** OR **#9**

**6,823**

**#9**

**'simulation based*'**

**4,276**

**#8**

**'simulation training'**/exp

**191,506**

**#7**

**#1** OR **#2** OR **#3** OR **#4** OR **#5** OR **#6**

**44,055**

**#6**

**'critical care'**:ab,ti

**21,695**

**#5**

**'emergency medicine'**:ab,ti

**4,101**

**#4**

**'crisis resource management'** OR **'advanced life support'**

**120,609**

**#3**

**'intensive care'**/de

**39,589**

**#2**

**'emergency medicine'**/de

**6,355**

**#1**

**'crisis intervention'**/exp

**Cinahl 17/12/19**

| **#** | **Query** | **Limiters/Expanders** | **Last Run Via** | **Results** |
| --- | --- | --- | --- | --- |
| S13 | S5 AND S8 AND S11 | Limiters - Published Date: 20090101-20201231 Expanders - Apply equivalent subjects Search modes - Boolean/Phrase | Interface - EBSCOhost Research Databases Search Screen - Advanced Search Database - CINAHL with Full Text | 153 |
| S12 | S5 AND S8 AND S11 | Expanders - Apply equivalent subjects Search modes - Boolean/Phrase | Interface - EBSCOhost Research Databases Search Screen - Advanced Search Database - CINAHL with Full Text | 180 |
| S11 | S9 OR S10 | Expanders - Apply equivalent subjects Search modes - Boolean/Phrase | Interface - EBSCOhost Research Databases Search Screen - Advanced Search Database - CINAHL with Full Text | 143,643 |
| S10 | Patient Care Team* OR Interprofessional Relation* OR team* OR interdisciplinary team* OR medical emergency team* OR medical emergency response team* | Expanders - Apply equivalent subjects Search modes - Boolean/Phrase | Interface - EBSCOhost Research Databases Search Screen - Advanced Search Database - CINAHL with Full Text | 139,880 |
| S9 | (MH "Interprofessional Relations+") | Expanders - Apply equivalent subjects Search modes - Boolean/Phrase | Interface - EBSCOhost Research Databases Search Screen - Advanced Search Database - CINAHL with Full Text | 27,914 |
| S8 | S6 OR S7 | Expanders - Apply equivalent subjects Search modes - Boolean/Phrase | Interface - EBSCOhost Research Databases Search Screen - Advanced Search Database - CINAHL with Full Text | 5,661 |
| S7 | simulation based* | Expanders - Apply equivalent subjects Search modes - Boolean/Phrase | Interface - EBSCOhost Research Databases Search Screen - Advanced Search Database - CINAHL with Full Text | 3,954 |
| S6 | Simulation train* | Expanders - Apply equivalent subjects Search modes - Boolean/Phrase | Interface - EBSCOhost Research Databases Search Screen - Advanced Search Database - CINAHL with Full Text | 2,714 |
| S5 | S1 OR S2 OR S3 OR S4 | Expanders - Apply equivalent subjects Search modes - Boolean/Phrase | Interface - EBSCOhost Research Databases Search Screen - Advanced Search Database - CINAHL with Full Text | 70,341 |
| S4 | "Crisis Resource Management" OR "advanced life support" OR "Emergency Medicine" OR "Critical Care" | Expanders - Apply equivalent subjects Search modes - Boolean/Phrase | Interface - EBSCOhost Research Databases Search Screen - Advanced Search Database - CINAHL with Full Text | 67,138 |
| S3 | (MH "Critical Care") | Expanders - Apply equivalent subjects Search modes - Boolean/Phrase | Interface - EBSCOhost Research Databases Search Screen - Advanced Search Database - CINAHL with Full Text | 20,607 |
| S2 | (MH "Emergency Medicine") | Expanders - Apply equivalent subjects Search modes - Boolean/Phrase | Interface - EBSCOhost Research Databases Search Screen - Advanced Search Database - CINAHL with Full Text | 10,813 |
| S1 | (MH "Crisis Intervention") | Expanders - Apply equivalent subjects Search modes - Boolean/Phrase | Interface - EBSCOhost Research Databases Search Screen - Advanced Search Database - CINAHL with Full Text | 3,314 |

**The Cochrane Library 16/12/19**

Date Run: 16/12/2019 21:23:05

ID Search Hits

#1 MeSH descriptor: [Emergency Medicine] this term only 255

#2 MeSH descriptor: [Crisis Intervention] explode all trees 154

#3 MeSH descriptor: [undefined] explode all trees 0

#4 ("Crisis Resource Management" OR "advanced life support"):ti,ab,kw (Word variations have been searched) 268

#5 ("Emergency Medicine" OR "Critical Care" OR "Critical Care"):ti,ab,kw (Word variations have been searched) 5564

#6 #1 OR #2 OR #3 OR #4 OR #5 5940

#7 MeSH descriptor: [Simulation Training] explode all trees 852

#8 (simulation based*):ti,ab,kw (Word variations have been searched)5759

#9 #7 OR #8 6243

#10 MeSH descriptor: [Interprofessional Relations] explode all trees 564

#11 MeSH descriptor: [Patient Care Team] explode all trees 1657

#12 (Patient Care Team* OR Interprofessional Relation* OR team* OR interdisciplinary team* OR medical emergency team* OR medical emergency response team*):ti,ab,kw (Word variations have been searched) 18888

#13 #10 OR #11 OR #12 19023

#14 #6 AND #9 AND #13 with Publication Year from 2009 to 2019, in Trials 77
